# Do we really need the arterial phase on CT in pelvic trauma patients?

**DOI:** 10.1007/s10140-020-01820-2

**Published:** 2020-07-19

**Authors:** Johannes Clemens Godt, Torsten Eken, Anselm Schulz, Kjetil Øye, Thijs Hagen, Johann Baptist Dormagen

**Affiliations:** 1 Division of Radiology and Nuclear Medicine, Oslo University Hospital Ullevål, Postboks 4956, Nydalen, 0424 Oslo, Norway; 2 Institute of Clinical Medicine, University of Oslo, Oslo, Norway; 3 Department of Anesthesiology, Oslo University Hospital Ullevål, Postboks 4956, Nydalen, 0424 Oslo, Norway; 4 Department of Diagnostic Physics, Oslo University Hospital Ullevål, Oslo, Norway

**Keywords:** Tomography, X-ray computed, Pelvic fractures, Extravasation of diagnostic materials, Angiography

## Abstract

**Purpose:**

To evaluate whether an arterial phase scan improves the diagnostic performance of computed tomography to identify pelvic trauma patients who received angiographic intervention on demand of the trauma surgeon.

**Methods:**

This retrospective single-center study was performed at an academic Scandinavian trauma center with approximately 2000 trauma admissions annually. Pelvic trauma patients with arterial and portal venous phase CT from 2009 to 2015 were included. The patients were identified from the institutional trauma registry. Images were interpreted by two radiologists with more than 10 years of trauma radiology experience. Positive findings for extravasation on portal venous phase alone or on both arterial and portal venous phase were compared, with angiographic intervention as clinical outcome.

**Results:**

One hundred fifty-seven patients (54 females, 103 males) with a median age of 45 years were enrolled. Sixteen patients received angiographic intervention. Positive CT findings on portal venous phase only had a sensitivity and specificity of 62% and 86%, vs. 56% and 93% for simultaneous findings on arterial and portal venous phase. Specificity was significantly higher for positive findings in both phases compared with portal venous phase only. Applying a threshold > 0.9 cm of extravasation diameter to portal venous phase only resulted in sensitivity and specificity identical to those of both phases.

**Conclusion:**

Arterial phase scan in addition to portal venous phase scan did not improve patient selection for angiography. Portal venous phase extravasation size alone may be used as an imaging-based biomarker of the need for angiographic intervention.

**Electronic supplementary material:**

The online version of this article (10.1007/s10140-020-01820-2) contains supplementary material, which is available to authorized users.

## Introduction

Pelvic fractures occur in 4–9.3% of patients with blunt trauma [1, 2]. Exsanguination remains a major challenge in treatment of these patients, with active hemorrhage resulting in a mortality rate of up to 40% [3–5]. Sources of bleeding are arteries, veins and venous plexus, or fractured cancellous bone [6–8]. With nonoperative management strategies becoming more important in trauma care, angiography with embolization is an accepted therapy for pelvic hemorrhage in hemodynamically stabilized patients [9–11].

Computed tomography (CT) is the preferred method to evaluate the need for pelvic angiography in addition to clinical parameters [8, 12, 13], with contrast extravasation (“blush”) being a strong indicator of active pelvic bleeding [14, 15]. According to a recent survey among level 1 trauma centers in the USA, 60% of participants scored contrast extravasation as an indication for angioembolization [16].

Although multiphase acquisitions in both arterial and portal venous phase CT have become standard in some trauma centers [17, 18], many institutions may still perform CT with a portal venous phase scan only [19]. In a multicenter study on pelvic trauma performed by Costantini et al., only 15.8% of all patients underwent CT including arterial phase scan [20]. CT in the arterial phase has been shown in some reports to be beneficial for identifying bleeding requiring treatment [18, 19], but minor hemorrhage can be difficult to be visualized in early arterial phase scanning [19]. Furthermore, some studies debate the clinical significance of contrast extravasation on CT because of its high false-positive rate [21, 22].

Radiation dose is another area of concern, since studies report a median age of pelvic trauma patients as low as 37 years [3, 23]. Data from the Norwegian National Radiation and Nuclear Safety Authority report a dose of 9.5 mSv when an additional CT scan is performed over the abdomen [24]. The potential benefit of an additional arterial phase should therefore outbalance the drawbacks of even a small increase of radiation exposure in trauma patient management [19, 25, 26]. The purpose of our study was to evaluate whether an arterial phase scan can improve the diagnostic performance to identify pelvic trauma patients who will need angiographic intervention.

## Material and methods

### Study design and setting

This retrospective study was performed at a Scandinavian regional trauma referral hospital for 2.9 million people, currently admitting approximately 2000 trauma patients per year. The project was approved by the hospital’s personal privacy ombudsman. Informed consent was waived because of the retrospective nature of the study.

Clinical data were obtained from the institutional trauma registry [27]. Anatomical injuries were coded according to the Abbreviated Injury Scale (AIS08) [28] by Association for the Advancement of Automotive Medicine (AAAM)–certified registrars utilizing all available information sources in the hospital’s clinical data systems. Injury severity score (ISS) [29] was chosen as a measure of overall injury. Inclusion criteria for the trauma registry are (i) all injured patients admitted with trauma team activation irrespective of ISS, (ii) penetrating injury proximal to elbow or knee, (iii) head injury with AIS severity code ≥ 3, and (iv) patients with ISS ≥ 10 admitted directly or via a local hospital less than 24 h after injury. Patients transferred ≥ 24 h after injury and those with an isolated single-extremity fracture are included only if they are received by a trauma team.

### Selection of participants

Patients > 16 years with pelvic fracture from blunt trauma between January 2009 and December 2015 were identified via the trauma registry. Inclusion criteria were available CT scan in arterial and portal venous phase, identified by the hospital’s RIS/PACS system (Syngo Studio V36, Siemens, Munich, Germany), and no pelvic surgery or angiography before CT. The CT protocol choice was made by the trauma surgeon based on the suspected trauma mechanism and initial examination. Angiographic intervention was requested by the trauma surgeon based on all available clinical and radiological data.

### Equipment and imaging protocol

CT examinations were performed on a 128-slice multidetector CT (MDCT) system (Somatom Flash, Siemens, Munich, Germany) with a collimation of 128 × 0.6 mm, or on a 64-slice MDCT system (Brilliance 64, Philips, Eindhoven, the Netherlands) with a collimation of 64 × 0.625 mm. Care Dose (Siemens) and Z-DOM (Philips) automatic exposure control calculated the correct dose according to patient size. The filter was standard, the matrix was 512, and the field of view was adjusted to patient size. Tube voltage was 120 kV; the field resolution was standard. Volume data sets were acquired, and axial, sagittal, and coronal reformations with 3-mm slice thickness were created.

Iomeprol 350 mg I/mL (Iomeron®, Bracco, Milano, Italy) intravenous contrast medium was administered for all examinations using a Medrad Stellant CT injection system (Bayer Healthcare, Whippany, NJ, USA) via an 18 G or larger peripheral venous access. A contrast dose of 2 mL contrast/kg body weight was administered followed by a 50 mL saline chase, both at a flow rate of 4 mL/s. Arterial phase scan was started by bolus tracking in the descending aorta. Portal venous phase scan was performed 65 s after the arterial phase scan, resulting in a delay of approximately 85 s.

### Image evaluation

Contrast extravasation and vascular injury were classified into six vascular territories modified after Hallinan et al. [12]. Contrast extravasation was defined as extravascular area of hyperattenuation [12, 30]. Hematoma was defined as fluid collection of an attenuation between 80 and 150 Hounsfield units (HU) in proximity to the pelvic fracture site.

Image evaluation was performed using the hospital’s PACS system. CT scans and angiography series were anonymized and read in a randomized order. The CT exams were prepared as one set containing the portal venous phase only and one set containing both arterial and portal venous phases. Two radiologists, each with more than 10 years of experience in trauma imaging, initially evaluated the exams independently before consensus was obtained for discordant findings. The consensus results were used for evaluation of the diagnostic performance. Both readers were blinded for any clinical information. The delay between reading the two sets was at least 6 weeks to avoid recognition bias [31]. Exams were considered positive if extravasation was identified on portal venous phase (first imaging set) or on both arterial and portal venous phases (second imaging set). Extravasation volume, location, attenuation in HU, and presence of visible direct vessel injury were registered. Maximum diameters of both the extravasation and the adjacent hematoma were measured on axial slices. Extravasation volume was assessed by using the “Region Growing” tool of the hospital’s image analysis software SyngoVia (Siemens, Munich, Germany).

### Reference standard

Angiographic intervention was the evaluated outcome (reference standard) for the two imaging sets. Angiography series were evaluated by a radiologist with more than 5 years of experience in interventional radiology for the presence and anatomic location of contrast extravasation, arterial injury, and type of intervention [12].

The principal investigator manually reviewed the patient’s medical records regarding conservative or surgical treatment.

### Statistical analyses

Wilcoxon and chi-squared tests were used for group comparisons. McNemar’s test with exact probability (binomial distribution) was used to determine differences in overall diagnostic performance, sensitivity, and specificity. Receiver operating characteristic (ROC) curve analyses were performed for comparisons of outcome vs. portal venous phase CT extravasation size and volume. Kappa values based on the reader’s evaluations prior to consensus were calculated to measure inter-observer agreement for contrast extravasation: < 0.20 = poor; 0.21–0.40 = fair; 0.41–0.60 = moderate; 0.61–0.80 = substantial; 0.81–1.00 = almost perfect [32]. Statistical analyses were performed using JMP version 11.2.1 (SAS Institute, Cary, NC, USA). For receiver operating characteristic (ROC) analyses, MedCalc version 19.2.1 (MedCalc, Ostend, Belgium) was used. A two-tailed *p* value of ≤ 0.05 was chosen to represent statistical significance.

## Results

During the study period, 169 patients with pelvic fracture underwent arterial and portal venous phase CT. Twelve patients were excluded due to pelvic surgery performed prior to CT. The remaining 157 patients (54 females and 103 males) were enrolled. Median age was 45 years (range 16–94); median ISS was 25 (range 4–75). Eighty-four patients (53.5%) underwent conservative treatment; 73 (46.5%) underwent orthopedic surgery. The 30-day mortality rate was 8.3% (13 patients). Table 1 summarizes clinical and laboratory characteristics for the 19 patients with CE in both arterial and portal venous phases, the 11 patients with CE in portal venous phase only, and the 127 without any CE on CT. Apart from ISS, no significant differences were observed between the groups.

**Table 1:** Clinical and laboratory characteristics of all patients

	CE in both arterial and portal venous phase	CE in portal venous phase only	No CE on CT	*p*
*n*	19	11	127	
ISS	33.1; 17.1 (24.9 to 41.3)*	23.3; 17.3 (11.6 to 34.9)	24.1; 14.4 (21.5 to 26.6)*	0.02*
SBP (mmHg)	109.1; 38.1 (90.7to 127.4)	121; 27.1 (103 to 139)	116.4; 25.3 (111.9 to 120.9)	n.s.
Glasgow Coma Score	11.5; 4.3 (9.4 to 13.6)	11.4; 4.8 (7.0 to 14.8)	12.6; 4 (11.8 to 13.2)	n.s.
Hemoglobin (g/dL)	12.4; 1.2 (11.8 to 13.0)	13.0; 2.3 (11.4 to 14.5)	12.9; 1.8 (12.6 to 13.3)	n.s.
Base excess (mmol/L)	− 6.2; 3.8 (− 8.1 to − 4.3)	− 5.2; 7 (− 10.4 to − 0.4)	− 4.8; 3.8 (− 5.4 to − 4.0)	n.s.
Lactate (mmol/L)	4.3; 3.2 (2.7 to 5.8)	2.9; 3.2 (0.4 to 5.6)	3.5; 2.6 (3.0 to 3.9)	n.s.

All values are presented as mean and standard deviation, with 95% confidence intervals in parentheses. *ISS* injury severity score, *SBP* systolic blood pressure, *CE* contrast extravasation. * = statistical significance

### Angiographic findings

**Fig. 1:**
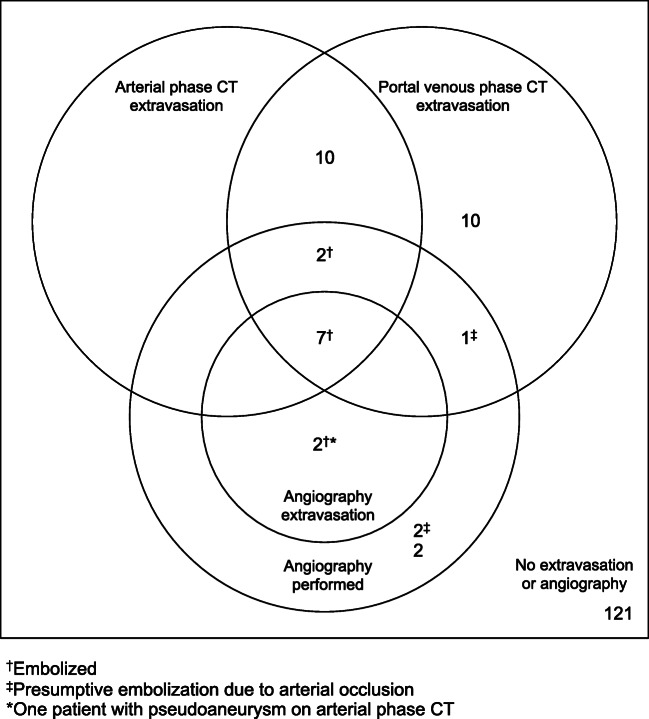
Contrast extravasation results of all patients, Venn diagram

Of the 16 patients who underwent angiography, 14 (88%) underwent embolization (Fig. 1). Nine of them had findings of contrast extravasation on CT in both arterial and portal venous phases, and one in portal venous phase only. Of the remaining six patients without any extravasation on CT, two showed findings of contrast extravasation on angiography and two underwent presumptive embolization due to occlusion. Vasospasm was the only angiographic finding in the remaining two patients, who did not receive embolization. A detailed summary of these results is displayed in Supplementary Table 4.

**Fig. 2:**
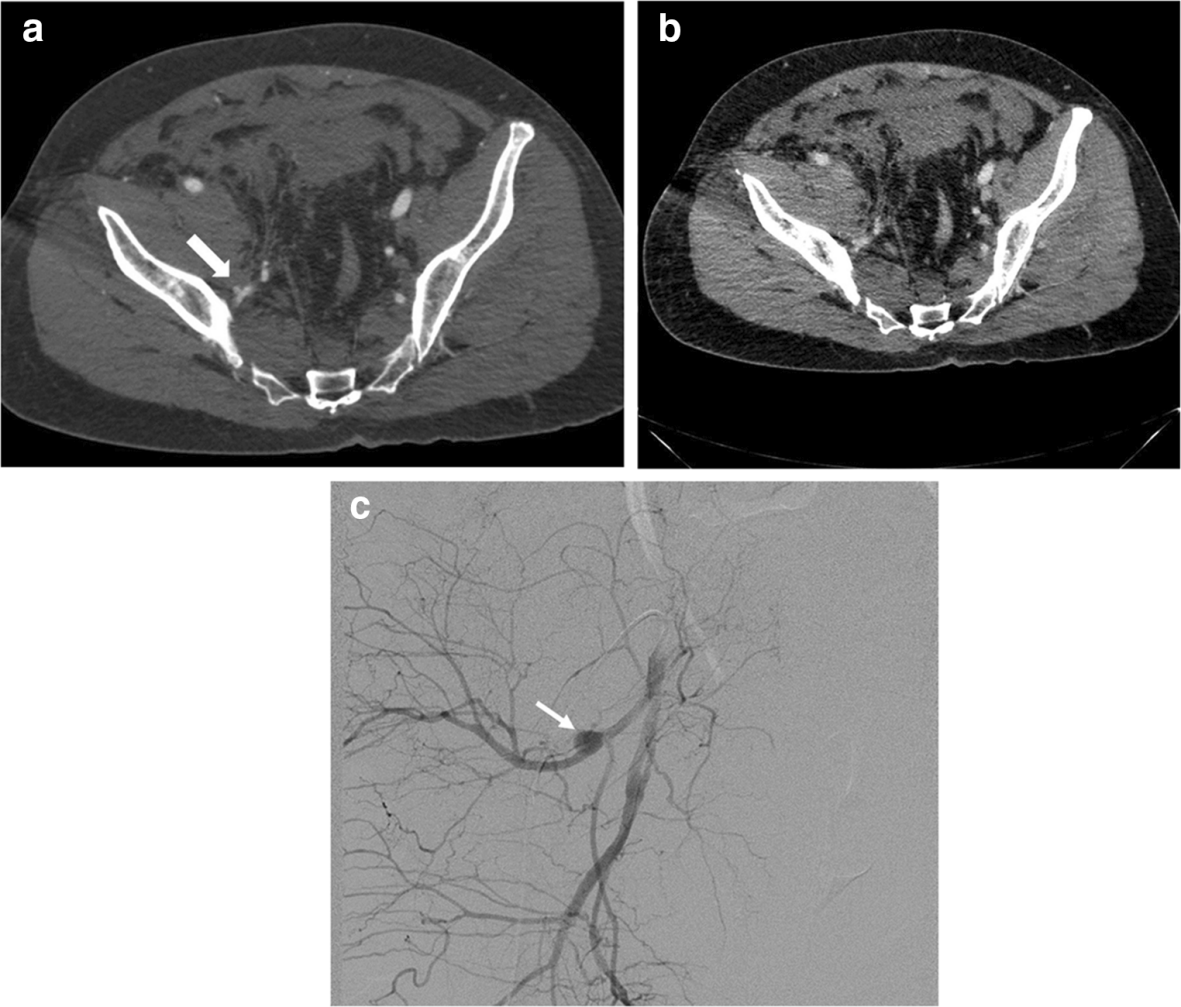
Pseudoaneurysm of the left superior gluteal artery visible on arterial phase CT ((**a**), white arrow), but not on portal venous phase CT (**b**). Corresponding angiography image ((**c**), white arrow)

In three patients, a pseudoaneurysm (PSA) was detected, one in the superior gluteal artery, visible on arterial phase CT and confirmed by angiography (Fig. 2); one in the inferior gluteal artery; and one in the internal pudendal artery. The latter two PSA were only detected on angiography; contrast extravasation on CT was found in both of these patients.

Patients who underwent angiography had significantly higher ISS, more deranged physiology, and higher 30-day mortality (Table 2).

**Table 2:** Clinical and imaging characteristics of all patients, grouped by angiography performed or not

	Angiography performed	Angiography not performed	*p*
*n*	16	141	
ISS	40.1; 16.7 (31.2 to 49.0)	23.4; 14.1 (21.0 to 25.7)	0.0002
SBP (mmHg)	85.1; 18.5 (75.4 to 94.8)	119.3; 25.9 (115.0 to 123.7)	0.0001
Glasgow Coma Score	10.5; 4.9 (7.8 to 13.1)	12.5; 3.9 (11.9 to 13.2)	0.04
Hemoglobin (g/dL)	11.5, 2.1 (10.4 to 12.6)	13.0; 1.7 (12.8 to 13.2)	0.003
Base excess (mmol/L)	− 6.4; 4.6 (− 8.8 to − 4.0)	− 4.8; 3.97 (− 5.5 to − 4.1)	0.01
Lactate (mmol/L)	5.2; 2.8 (3.6 to 6.7)	3.4; 2.6 (2.9 to 3.8)	0.002
30-day mortality	25 (7.3 to 52.4)	6.4 (3.0 to 11.8)	0.02

All values are presented as mean and standard deviation, with 95% confidence intervals in parentheses. 30-day mortality is given as percent with 95% intervals. *ISS* injury severity score, *SBP* systolic blood pressure, *GCS* Glasgow Coma Scale

### Imaging findings on computed tomography

**Fig. 3:**
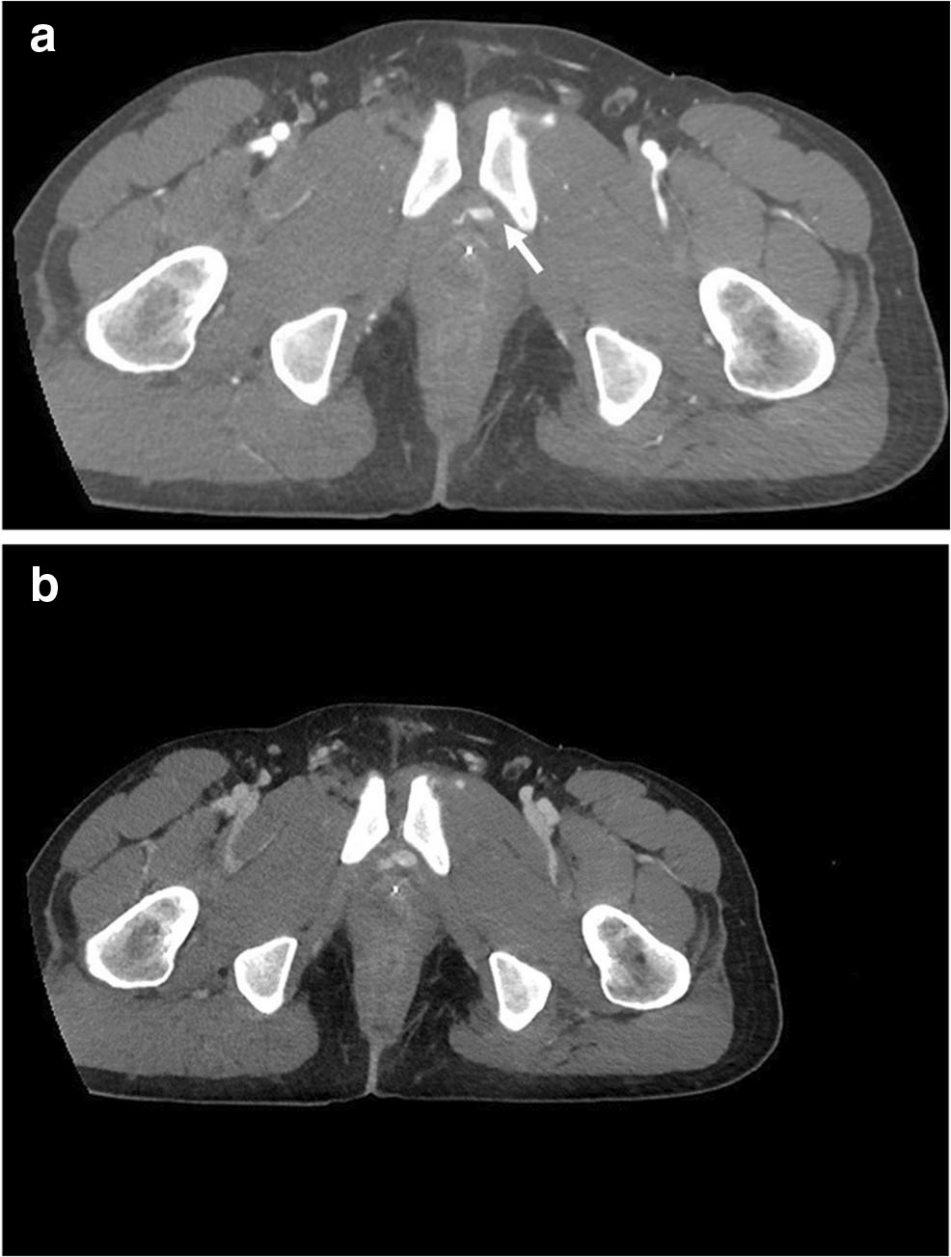
Extravascular area of hyperattenuation from the left pudendal vascular territory (white arrow) in arterial phase (**a**) and portal venous phase (**b**) CT

**Fig. 4:**
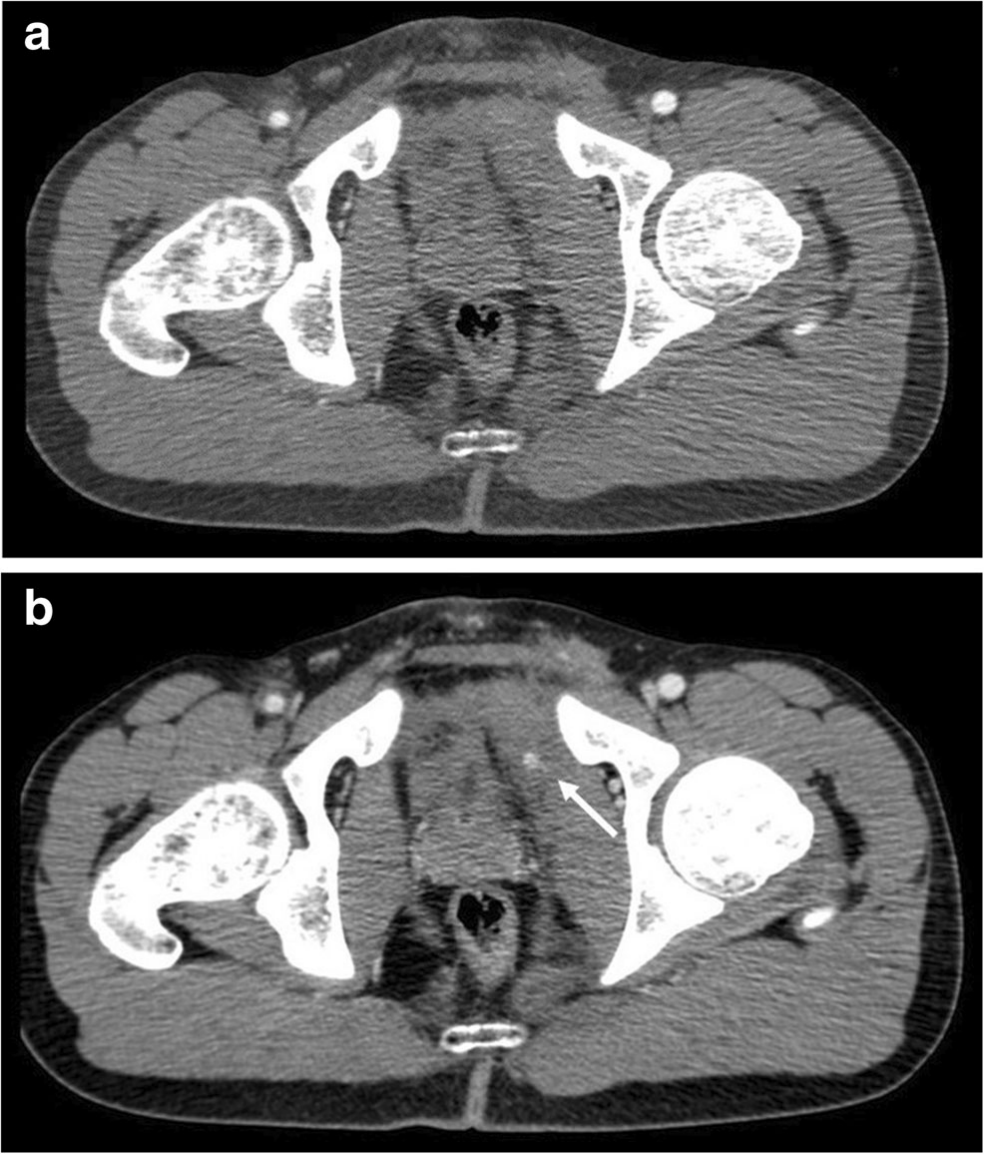
No findings of extravasation in arterial phase CT (**a**). Extravascular area of hyperattenuation from the left pudendal vascular territory (white arrow) in portal venous phase only (**b**)

Contrast extravasation on CT was found in 30 (19%) of the enrolled patients, 19 (63%) in both arterial and portal venous phase (Fig. 3), and 11 (37%) in portal venous phase only (Fig. 4).

Extravasation was located in the anterior internal iliac territory in 24 patients (80%), in the posterior internal iliac territory in five patients (17%), and in the presacral vascular territory in one patient (3%). No patients had extravasation in more than one vascular territory. The mean extravasation size was 0.9 cm in the arterial phase and 1.5 cm in the portal venous phase.

The radiologists agreed in 135 of 157 cases (86%), and the inter-observer agreement was moderate (kappa = 0.58).

Extravasation size (1.5 vs. 0.8 cm, *p* = 0.02) and volume (3.5 vs. 0.6 mL, *p* = 0.02) on portal venous phase CT were larger in patients with concomitant arterial phase extravasation also in arterial phase, compared with patients with portal venous extravasation only. No significant differences were observed for hematoma size and attenuation of extravasation and hematoma between the two patient groups. In addition, portal venous phase extravasation size (1.9 vs. 0.9 cm, *p* = 0.02) and volume (3.8 vs 1.8 ml, *p* = 0.02) were larger in patients who underwent angiography compared with those who did not undergo angiography.

### Diagnostic performance of additional arterial phase CT

When considering referral to angiographic intervention as outcome, demanding CE on both arterial and portal venous phase CT vs. only on portal venous phase CT changed the overall diagnostic performance (*p* = 0.01, McNemar’s test; Table 3) by increasing specificity from 86 to 93% (*p* = 0.002) without changing sensitivity (from 63 to 56%, *p* = 1.0). ROC analysis showed that a sensitivity of 56% with a specificity of 93% could also be obtained if a portal venous CE diameter of > 0.9 cm was the only criterion for referral. Supplementary Table 5 shows sensitivity and specificity at different cutoff thresholds for portal venous phase extravasation size. Using a portal vein extravasation volume of > 0.63 mL resulted in almost identical results (sensitivity 56%, specificity 92%). Area under the curve (AUC) for the two ROC curves was 0.77 and 0.76 respectively. A post hoc power calculation for AUC showed that our study had sufficient power to detect changes of AUC by 0.2.

**Table 3:** Diagnostic performance of portal venous and combined arterial and portal venous phase CT regarding the need of angiographic intervention

	Portal venous phase CT		Arterial and portal venous phase CT	
	*n*	% (95% CI)	*n*	% (95% CI)
Sensitivity	10/16	62.5 (35.4 to 84.8)	9/16	56.2 (29.9 to 80.2)
Specificity	121/141	85.8 (78.9 to 91.1)	131/141	92.9 (87.3 to 96.5)
PPV	10/30	33.3 (22.3 to 46.6)	9/19	47.4 (30.1 to 65.3)
NPV	121/127	95.3 (91.4 to 97.4)	131/138	94.9 (91.5 to 97.0)

A positive test was defined as any contrast extravasation in the arterial phase and portal venous phase when both sets were read and any extravasation in the portal venous phase when only portal venous phase imaging was read. *PPV* positive predictive value, *NPV* negative predictive value

## Discussion

Identifying patients at risk for severe hemorrhage after pelvic trauma is critical [20]. In addition to clinical signs and laboratory markers indicating hemorrhagic shock, CT findings of ongoing bleeding are important for decision-making and prompt intervention [9]. In fact, the reported use of CT in patients with pelvic fracture and signs of hemorrhagic shock is 85% [20].

The identification of extravasation on CT indicates a major bleeding from an arterial or venous injury, but in the current relevant literature, the exact vascular source is not always clearly described [9, 21]. From the point of view of the radiologist and the surgeon, it is important to identify the source of pelvic bleeding on CT because arterial, venous, and cancellous bone hemorrhage needs different treatment. Arterial injuries are especially critical because they are more severe and life-threatening and can effectively be treated with angiographic intervention [32].

Attempts have been made to diagnose pelvic arterial injuries on CT angiography [18, 20, 33] by adding an arterial phase to the more widespread portal venous phase CT scan [20]. Arterial injuries are seen as extravasation on arterial imaging or as increasing hematomas on delayed imaging, whereas venous injuries would lack contrast extravasation in the early arterial scanning [33].

In the present study, we wanted to determine if there is a diagnostic benefit of an added arterial scan for predicting the decision of referral for angiographic intervention. We therefore compared a single portal venous imaging set with a combined set of arterial and portal venous phases.

### Angiographic intervention

The fraction of patients who underwent angiography was 10%, comparable with the US multicenter study by Coccolini et al. (9.6%) [34]. We experienced a high yield of angiography, leading in 14 out of 16 cases (88%) to embolization, which is in line with the studies by Yuan et al. (96%) and Brun et al. (88%) [9, 35].

The surgeon’s decision to refer to angiography at our hospital is based on a combination of continuous blood loss, clinical signs of ongoing bleeding, and extravasation on CT [36]. Other institutions propose relative hypotension with a systolic blood pressure decrease of 30 mmHg to identify patients with need for angiography despite no contrast extravasation seen on CT [37]. The Eastern guidelines recommend to consider all patients older than 60 years with major pelvic fractures for angiography, regardless of hemodynamic status [38]. As our trauma surgeons do not apply strictly quantitative thresholds of biomarkers, this may have led to a lower number of negative angiographies in our study population.

In concordance with other studies, clinical and laboratory characteristics such as ISS, systolic blood pressure, blood gas analysis results, and mortality differed significantly between patients referred to angiography and those without angiography referral [9, 10].

### Imaging findings on computed tomography

In the literature, the fraction of patients with contrast extravasation on CT in pelvic trauma varies between 15 and 23% [9, 37, 39], similar to our study (19%). It has been shown in several studies that not all patients with contrast extravasation on CT require angiography [22, 39]. This was also observed in our study population, where 33% of patients with CT extravasation were referred to angiographic intervention (10/30).

Only a few studies distinguish between arterial and portal venous phase CT in pelvic trauma. Anderson et al. evaluated extravasation foci and vascular injuries in 53 patients in arterial, portal venous, and delayed phase [33]. Fu et al. assessed the benefit of additional arterial phase CT in the evaluation of arterial hemorrhage in 144 pelvic trauma patients. Compared with our material (12%), the fractions of patients with extravasation in the arterial phase were higher in these studies (31% and 28%, respectively). These differences are probably explained by differences in patient selection to undergo CT in both arterial and portal venous phases between the studies. Furthermore, the numbers of patients with extravasation on either arterial or portal venous phase who received angiographic intervention were also different, presumably due to local treatment procedures.

Indication for angiographic intervention may differ, and up to 40% of patients with extravasation on arterial phase scanning will not need angiography [39]. The increased sensitivity of submillimeter slice CT techniques in recent years enables detection of insignificant microbleeds [39] sometimes precluding the need for angiography. In our study, nine out of nineteen patients with extravasation in both arterial and portal venous phases underwent angiographic intervention, compared with only one out of eleven with extravasation in portal venous phase only. The latter patient was embolized despite the absence of extravasation at angiography. A similar finding was reported in a study by Fu et al. where no active bleeding was observed on angiographic intervention in 4 of 5 cases with portal venous phase extravasation only. Therefore, extravasation on arterial phase might indicate a more severe bleeding.

In our study, arterial phase CT showed a PSA in one patient. Imaging in portal venous phase alone would not have depicted this injury (Fig. 2). This patient was hemodynamically unstable, but did not have findings of contrast extravasation on CT. Traumatic PSA after pelvic trauma are not common, but treatment is of importance to prevent delayed hemorrhage [19].

Contrast extravasation on arterial phase CT is widely accepted as indicative of arterial hemorrhage, whereas contrast extravasation on portal venous phase alone is generally considered to be caused by venous bleeding (Fig. 4) [33, 40–42]. Such patients may have arterial bleeding from small arterial branches with extravasation too small to be seen on arterial phase CT, but it is assumed that these minor hemorrhages will not cause severe hemorrhagic hypotension and therefore should not lead automatically to angiography [19].

### Comparison of CT and angiographic findings

Three of our patients had negative angiography after contrast extravasation seen on CT, one on portal venous phase only and two in both phases. Nevertheless, all three patients were embolized. Yuan et al. describe this as a clinical dilemma [35]. Spontaneous endogenous hemostasis could be an explanation for this discrepancy in our patients. Absence of contrast extravasation on CT does not always exclude the need for angiography, as seen in six patients in our study (Fig. 1) [10, 34]. Indication for angiographic intervention without signs of CT extravasation include direct vessel injuries, like PSA, arteriovenous fistula, and vessel occlusion [26].

Our two patients with extravasation on angiography and negative CT were both older than 60 years. In the first patient, indication for angiography was a PSA in the superior gluteal artery visible on arterial phase CT. The other patient had angiography performed at a time interval of more than 5 h after a negative CT, due to increasing hemodynamic instability. Such discrepancy between extravasation seen on CT and angiography can be related to vessel spasm after secondary local inflammatory response in patients with bleeding or hypotension [37, 43]. According to Dreizin et al., arterial bleeding can be occult at CT in 20–40% of patients [41, 44].

In addition to the one aforementioned PSA depicted on arterial phase, two more cases of PSA, both under 5 mm in diameter, were seen on angiography but not depicted on CT neither in arterial nor portal venous phase. These were located in the region of contrast extravasation on CT, so they may have been hidden by extravasated contrast.

Occlusion and spasm of small injured arterial branches may be difficult to depict on CT, and extrinsic compression by increasing surrounding hematoma in the time interval between CT and angiography can also occur.

### Additional arterial phase CT

Combined arterial and portal venous CT did not increase diagnostic performance compared with portal venous phase CT alone, but increased the specificity from 86 to 93%, which would result in a lower rate of unnecessary angiographies. Fu et al. also showed that differentiation between arterial and venous bleeding was possible based on multiphasic CT, but this did not affect patient treatment [19]. Yoshikawa et al. reported similar diagnostic performance for angioembolization based on contrast extravasation on CT, with 57% sensitivity and 86% specificity. This was comparable to our findings, but Yoshikawa et al. did not differentiate between arterial and portal venous phase [45].

Several groups have reported quantitative measurements for treatment decision. According to Murakami et al., size of portal venous extravasation can help determine clinically relevant hemorrhage and predict successful treatment [46]. In a study with 21 pelvic, 11 soft tissue, and 48 abdominal parenchymal organ injuries, Michailidou et al. proposed a value of 1.5 cm extravasation diameter as threshold for hemorrhages requiring intervention [21]. For the arterial phase, Ramin et al. determined the need for angiography with 100% sensitivity and 62% specificity at a threshold value of 20 mm^2^ arterial phase extravasation size [22]. This value corresponds approximately to a circle of 0.5 cm in diameter.

In our study, no additional value of arterial phase scan was seen when the extravasation size threshold on the portal venous phase was 0.9 cm. A similarly high negative predictive value of 95% was achieved with both protocols (Table 3), providing reassurance that in the absence of contrast extravasation, the pelvis is unlikely to be the source of hemorrhagic shock [39]. Further, areas under the ROC curves for venous phase extravasation size and volume were almost identical when predicting need of angiographic intervention. Therefore, we suggest portal venous phase extravasation size as the preferred measurement method since it is fast and easy to acquire.

Our study is not without limitations. During the study period, not all patients with pelvic injury underwent both arterial and portal venous phase CT. The fact that imaging protocol was assigned based on initial evaluation by the attending surgeon might have led to inclusion bias; however, dual-phase CT was assigned to patients with assumed higher probability for pelvic hemorrhage, thereby increasing the probability of revealing a significant difference between the two imaging sets.

The number of patients who underwent angiography in our study population was relatively small and a larger study population might be able to depict minor significant differences.

Due to the retrospective nature of our study, we could not determine the degree to which the attending surgeon was influenced by extravasation on CT when deciding to refer for angiographic intervention. However, since not all patients with extravasation on CT were referred for angiographic intervention, we assume decisions were based on a relatively balanced evaluation of all available relevant clinical and radiological information by the surgeon.

In conclusion, an additional arterial phase scan seems not to improve patient selection for angiography. The only benefit of arterial phase CT was a slightly higher specificity and the detection of rare arterial injury, such as pseudoaneurysm. Portal venous phase scanning alone could detect all areas of contrast extravasation and performed equally well if quantification of contrast extravasation diameter or volume was included. We therefore suggest that the diameter of extravasation on portal venous phase alone can be used as a decisive imaging-based biomarker of the need for angiographic intervention.

## Electronic supplementary material

ESM 1 (DOCX 40 kb)

ESM 2 (DOCX 26 kb)

## Data Availability

The data that support the findings of this study are available from the Oslo University Hospital, but restrictions apply to their availability. The data belong to OUH, and Norwegian legislations regarding hospital-owned data apply. The data were used under license for the current study and so are not publicly available.
