# Supplementary material for: Do we really need the arterial phase on CT in pelvic trauma patients?
Source: Emerg Radiol. 2020 Jul 19;28(1):37–46. doi: 10.1007/s10140-020-01820-2 (PMC7835176; doi:10.1007/s10140-020-01820-2)
Supplement: Supplementary file 1 [file 10140_2020_1820_MOESM1_ESM.docx]

Table 4 (supplement) : Results overview for contrast extravasation in CT.

n (157)	Extravasation on arterial phase CT	Extravasation on portal venous phase CT	Angiography performed	Extravasation on angiography	Embolized
121	no	no	not performed	n.a.	n.a.
2	no	no	yes	no	yes^
2	no	no	yes	no	no
7	yes	yes	yes	yes	yes
2	yes	yes	yes	no	yes
2	no*	no	yes	yes	yes
10	yes	yes	not performed	n.a.	n.a.
10	no	yes	not performed	n.a.	n.a.
1	no	yes	yes	no	yes^

* one patient with findings of pseudoaneurism (PSA) on arterial phase CT.

^presumptive embolization due to arterial occlusion.

n.a.=not applicable
