# Supplementary material for: Do we really need the arterial phase on CT in pelvic trauma patients?
Source: Emerg Radiol. 2020 Jul 19;28(1):37–46. doi: 10.1007/s10140-020-01820-2 (PMC7835176; doi:10.1007/s10140-020-01820-2)
Supplement: Supplementary file 2 [file 10140_2020_1820_MOESM2_ESM.docx]

**Table 5 (supplement): Sensitivity/specificity at different cutoff thresholds for portal venous phase extravasation size regarding angiography performed or not.**

| Extravasation size in portal venous phase, cm | Sensitivity % (95% CI) | Specificity % (95% CI) |
|---|---|---|
| ≥0 | 100.0 (79.4 to 100.0) | 0.0 (0.0 to 2.6) |
| >0 | 62.5 (35.4 to 84.8) | 86.5 (79.8 to 91.7) |
| >0.4 | 62.5 (35.4 to 84.8) | 87.2 (80.6 to 92.3) |
| >0.5 | 56.2 (29.9 to 80.2) | 90.1 (83.9 to 94.5) |
| >0.9 | 56.2 (29.9 to 80.2) | 92.9 (87.3 to 96.5) |
| >1 | 43.7 (19.8 to 70.1) | 94.3 (89.1 to 97.5) |
| >1.1 | 43.7 (19.8 to 70.1) | 95.0 (90.0 to 98.0) |
| >1.2 | 31.2 (11.0 to 58.7) | 95.4 (91.0 to 98.4) |
| >2.13 | 31.2 (11.0 to 58.7) | 100.0 (97.4 to 100.0) |
| >3.75 | 0 (0.0 to 20.6) | 100.0 (97.4 to 100.0) |
